# Supplementary material for: Behavioral Welfare Research for the Management of Sanctuary Chimpanzees (Pan troglodytes)
Source: Animals (Basel). 2023 Aug 11;13(16):2595. doi: 10.3390/ani13162595 (PMC10451351; doi:10.3390/ani13162595)
Supplement: Supplementary file 1 [file animals-13-02595-s001.zip › animals-2383262-supplementary.pdf]

Supplement S1. Social monitoring system ethogram.

Table S1. Behavior name, type of behavior, modifiers if included, and definitions of behavior.

Behavior	Type	Modifiers	Definition
**Inactive**	Interval	N/A	Chimpanzees are not moving and not active in any other behavior. This includes instances during which a chimpanzee holds an object without actively manipulating it or carrying it, behaviors such as resting with a blanket on, touching an object while resting (such as sitting on a spool) but not actively using (nesting, chewing, manipulating, etc.) the object, resting (which may or may not be "sleeping") and resting alert. This also includes watching people and chimps.
**Pant Hoot**	Interval	N/A	Defined in **Nishida et al. 2010** as having four stages: introduction, build-up, climax, and let-down" and used in a variety of situations including but not limited to arriving at a food source, greeting subordinate (with pants and pant grunts), as part of display, as a threat or to let neighbors know their presence.
**Other**	Interval	Give, Receive, Mutual, Unknown	Behavior not otherwise listed, which includes unknown behaviors, urinating, and defecating. Can include behaviors that may seem abnormal but have not been defined/specified.
**Food/Drink**	Interval	N/A	Behavior in which the focal is eating (chewing and/or swallowing), wadging (i.e., with biscuits and/or produce), foraging behaviors (searching and move materials to look for bits of food on the ground) and any drinking, including pools or sucking on or chewing ice. Includes food-based enrichment related behaviors such as using the puzzle feeders and devices. Includes food-based vocalizations such as food grunts.

**Locomote**	Interval	N/A	Behavior in which the focal is actively moving. Includes knuckle walking, brachiating, bipedal walking, climbing structure, mesh and/or tree, crawling, running, wheel barrowing, swinging.
**Self**	Interval	N/A	Behavior in which the focal is focused on themselves. Includes self-directed, scratching, self-grooming, masturbation and/or play with self.
**Object Manipulation**	Interval	N/A	Behavior includes environment or non-food enrichment, including nesting with objects, carrying or mouthing objects such as plastics, cloth, stuffed animals, etc., including mouthing or chewing on objects/environment and playing with items such as blankets.
**Engage**	Interval	N/A	Behavior in which the focal is focusing their attention on people, not chimpanzees or self. Includes throwing at people, alarm barking at new people, following people, playing with people, etc. "People" is defined as any human primate. This includes but is not limited to any staff members of Chimp Haven, the public (anyone who is visiting on site but is not on staff or an intern) and other workers on site such as construction. It does not include just glancing, looking or watching people. This is an active behavior in which the animal is directing behaviors at a person such as displaying, spitting, throwing feces, eliciting play, panting, bobbing, etc.
**Tandem Walk**	Interval	Give, Receive, Mutual, Unknown	Tandem Walk: Walking while holding another chimpanzee, "train".
**Tandem Rock**	Interval	Give, Receive, Mutual, Unknown	Rocking while holding another chimpanzee.

**Hair Plucking or Pulling (Social)**	Interval	Give, Receive, Mutual, Unknown	Pulling hair out of another chimpanzee.
**Floating Limb**	Interval	N/A	Individual's limbs (usually leg and foot or arm) slowly lifts upward toward the shoulder - the chimpanzee appears not to notice limb movement and may even be startled by its sudden appearance.
**Self-Aggression**	Interval	N/A	Violence directed at self, may be hitting, biting or other, may occur following a floating limb when an animal attacks a limb as it is not a part of its own body. Behavior does not break the skin.
**Self-Injurious**	Interval	N/A	Violence directed at self that results in wounds or more permanent damage to body.
**Hair Plucking/Pulling/Eating**	Interval	N/A	Manipulating hair by extraction, not the same as moving hair around while self-grooming, can include ingesting hair.
**Rocking**	Interval	N/A	Rhythmic body motion back and forth in one direction, must be more than approximately 3 inches in degree, more severe rocking includes a noise associated.
**Poke Eye/Body**	Interval	N/A	Putting finger into the eye socket or body part, not grooming.
**Wound Picking**	Interval	N/A	Excessive attention to an existing wound that was not received as self injurious behavior, including removing more skin or muscle tissue than was originally noted from a wound report or exam from vet staff.
**Self-Suck**	Interval	N/A	Sucking on self for an extended period of time.
**Head toss**	Interval	N/A	Circular movement of the head.
**Coprophagy**	Interval	N/A	Ingesting feces.
**Drink Urine**	Interval	N/A	Ingest urine from self or other.
**Feces Painting**	Interval	N/A	Smearing feces on environmental surface.

**Regurgitate & Re-ingest**	Interval	N/A	Coughing up already swallowed food, may also re-ingest the food.
**Submissive**	Interval	Give, Receive, Mutual, Unknown	Any behavior in which the focal individual is either giving or receiving a submissive behavior. "Submissive" is defined as any behavior in which the individual is acquiescing or appeasing another individual. Includes wrist present, avoid (meaning an individual displaces or is displaced by another that is approaching), fear grimace (lips pulled back revealing teeth, accompanied sometimes with a high pitched whine), bow/bob (the body of the individual is moving up in down, usually backing up from the other), and pant/pant-grunt (breathy sound from the air sacs, sometimes with a grunting noise and directed toward a higher ranking individual).
**Sexual**	Interval	Give, Receive, Mutual, Unknown	Any behaviors involving the anogenital region between partners. Includes sexual present, sexual solicitation, copulation. Does not include masturbation or social mounting.
**Groom**	Interval	Give, Receive, Mutual, Unknown	Focal individual picks through the hair or removes debris from the skin of one other individual using hands and/or mouth, or an object (i.e. comb or stick).
**Play Related**	Interval	Give, Receive, Mutual, Unknown	Relaxed face, open mouth (sometimes), relaxed body posture, voluntary, incompletely functional (does not achieve a specific survival goal such as eating), repeats without being abnormal, can include chase, tickle, foot-pull game, etc.
**Oral**	Interval	Give, Receive, Mutual, Unknown	Mouthing and kissing behaviors in which another group member is in contact with the face of a partner in a positive social interaction.
**Embrace**	Interval	Give, Receive, Mutual, Unknown	Behaviors that are usually observed as a full body hug either from the side (around the back of a partner) or face-to-face clasping together.

**Other**	Interval	Give, Receive, Mutual, Unknown	Behaviors that include reach out, touch, mount, food share, walk together and follow.
**Aggressive**	Interval	Give, Receive, Mutual, Unknown, Contact, No Contact, and Unknown	Any behaviors in which the focal is involved in an encounter in which hostile or violent behavior is involved. Examples include swagger, lunge, hit at, charge, chase, steal item, hit, kick, push, stomp, grapple, bite.
**Display**	Interval	Give, Receive, Mutual, Unknown	Chimpanzee typically has pilo-erect hair, is usually pant hooting, throwing objects and running or charging. Does not always have a clear recipient or clear motivation (can be excitement or aggression).
**Out of View**	Interval	N/A	Whenever the focal animal is not visible and information such as behavior and proximity cannot be established.
**Group Aggression**	All Occurrence	N/A	A whole or majority of the group is fighting. Usually includes many aggressive behaviors in quick succession between multiple group members.
**Abnormal**	All Occurrence	N/A	Any group member other than the focal is exhibiting abnormal behavior.
**Display**	All Occurrence	N/A	Any group member other than the focal displays but not during or on a scan.

**Correlative Behaviors**	All Occurrence	Extra Group Aggression, Shifting, Human Interaction, Provision, Disturbance	**Extra-group aggression**: groups across the hall or nearby can and will fight/display/food bark and this will likely change the behaviors of the groups in the immediate vicinity, which then may cause the focal to change their behavior in response.<br>**Shifting:** the period of time in which the group is being shifted away from one part of their enclosure, usually for cleaning, can include food distribution. Includes a change in the available space during the focal scan.<br>**Human Interaction:** Any person that attempts or succeeds in directly interacting with any member of the group. Includes interaction time. Excludes any interaction involving food.<br>**Provision:** Caregiver, vet tech or enrichment tech gives the group food, including forage, biscuits, enrichment, medications, or juice. Includes training sessions.<br>**Disturbance:** Any kind of unusual incident that could alter the behavior of the chimpanzees such as loud noises, sudden changes in the environment (power outage), unusual people or vehicles within the vision or earshot of the chimps, anything not already covered.
**Affiliative**	All Occurrence	Give, Receive, Mutual, Unknown	Any affiliative behavior between any group members, not occurring during a scan.
**Sexual**	All Occurrence	Give, Receive, Mutual, Unknown	Any behaviors involving the anogenital region between partners that does not occur during a scan. Includes sexual present, sexual solicitation, copulation. Does not include masturbation or social mounting.
**Proximity**		None – no other chimps are in proximity of the focal.	Any chimpanzee within 1 meter (3 ft) of the trunk of another chimpanzee is considered within proximity. The animals for each group will be listed here to use as proximity partners. If unknown or unclear, do not enter a proximity.
